# Health literacy: association with socioeconomic determinants and the use of health services in Spain

**DOI:** 10.3389/fpubh.2023.1226420

**Published:** 2023-10-12

**Authors:** Nayara Tamayo-Fonseca, Pamela Pereyra-Zamora, Carmen Barona, Rosa Mas, Mª Ángeles Irles, Andreu Nolasco

**Affiliations:** ^1^Research Unit for the Analysis of Mortality and Health Statistics, Department of Community Nursing, Preventive Medicine, Public Health and History of Science, University of Alicante, Alicante, Spain; ^2^Conselleria de Sanitat Universal i Salut Pública, Generalitat Valenciana, Valencia, Spain; ^3^Foundation for the Promotion of Health and Biomedical Research in the Valencian Region (FISABIO), Valencia, Spain; ^4^CIBER de Epidemiología y Salud Pública (CIBERESP), Madrid, Spain

**Keywords:** health literacy, HLS-EU-Q16, use of health services, health inequalities, Spain, health promotion, socioeconomic determinants, new health technologies

## Abstract

**Background:**

Health literacy (HL) is the set of social and cognitive skills that determine person’s level of motivation and the ability to access, understand and use information to promote and maintain good health. The aim of this study is to assess the level of health literacy, and to analyze its relationship with sociodemographic variables, state of health, and use of health services in the population aged 15 and over in the Valencian Community (Spain).

**Methods:**

Cross-sectional study based on a sample of 5,485 subjects participating in the Health Survey of the Valencia Community. The HLS-EU-Q16 was used. As outcome variables we considered HL categorized into 2 levels: Inadequate or Problematic HL and Sufficient HL and the standardized literacy index. Prevalence rates and HL means were estimated and OR were calculated to analyze the association between variables.

**Results:**

A total of 12.8% of the subjects surveyed presented an inadequate or problematic degree of HL. This percentage was higher in people >85 years (63.1%), with a low level of education (46.5%), in retired people (27.4%) or in other work situations (25.0%), in foreigners (18.1%), in low-income people (16.2%), with a perception of poor health status (26.9%), chronic disease (18.5%) or with activity limitations (56.4% severe, 19.7% not severe). Significant differences were found. With the exception of chronic disease, all the variables analyzed were associated with HL. Low HL was associated with a lower consumption of medicines, a greater use of health services, general medical consultations, poorer knowledge of new health technologies and fewer preventive health visits.

**Conclusion:**

The percentage of inadequate or problematic HL was globally not very high, but certain population subgroups notably presented a high degree of inadequate or problematic HL. Raising the HL level of such groups should be regarded as a priority. HL was shown to be associated with the service use and new health technology use. Enhancing the population’s HL should lead to the following: a greater probability of adopting preventive practices; improving the use of the health system; and boosting people’s abilities to manage and to improve their own health.

## 1. Introduction

Health literacy (HL) is the set of social and cognitive skills that determine a person’s degree of motivation and ability to access, understand and use information to promote and maintain good health (1).

In recent years, there has been an increasing amount of research on health literacy. Recent studies have described the essential role of HL as it is one of the major predictors of the population’s health status. HL is also recognized as a key factor in the reduction of health inequalities (2–14).

Several institutions have been recommending the performance of standardized and regular measurements of the general population’s HL in order to broaden the evidence and to implement public health policies allowing people to promote and maintain their own good health (10, 14, 15).

Several instruments can be used to assess HL (7, 16–18). They are based on different definitions, conceptual frameworks and they evaluate distinct dimensions, skills, and domains. Europe has been developing instruments to measure HL since 2009 and they have been designed by the European Consortium on Health Literacy (HLS-EU Consortium) – which is part of the European Commission’s European Public Health Programme –, and since 2018, by the HLS_19_ Consortium of the WHO Action Network on Measuring Population and Organizational Health Literacy (M-POHL), which is integrated in the European Health Information Initiative (EHII WHO-Europe) (19, 20).

The first European Health Literacy Survey (HLS-EU) was conducted within the HLS-EU Consortium project, which was based on a comprehensive and general HL conceptual framework and definition. The project generated a range of HL measurement instruments for the general population, among which a questionnaire with 47 items (HLS-EU-Q47) that fall into 4 information management dimensions (find information, understand it, evaluate it and apply it) linked to each of the 3 health domains (care, prevention and health promotion). A short form of the HLS-EU-Q47 was also developed called the HLS-EU-Q16 questionnaire, with 16 items (20). In Spain, the latter has proven to be a swift, adequate, and valid instrument to measure the population’s level of HL (21).

In Spain, few studies have analyzed the HL of the general population (22, 23). The results of the first European Health Literacy Survey conducted between 2009 and 2012 showed that results varied significantly among different countries. In Spain, at least half of the respondents presented limited health literacy (with inadequate or problematic levels), but the average HL level was slightly higher than the average of the other European countries that took part in the survey (9, 20).

Various socioeconomic determinants affecting HL have generally been described. Education has shown to be a significant predictor of HL, together with age, employment and social status, income/financial deprivation, etc. (11, 20, 24, 25).

Previous studies have shown a range of negative effects on healthy lifestyles, self-perceived health, and activity limitations among people with limited HL (20, 25). Thus, patients with chronic diseases also present greater complications and vulnerability in their disease management (10).

Among older people, HL has been associated with poorer overall health, higher mortality rates, later diagnoses and increased premature mortality in the case of certain cancers (10, 17, 26–28).

Regarding service use, low HL has been associated with a higher number of hospitalizations and emergencies, a lesser use of preventive services, and a worse ability to take medication appropriately and to correctly interpret health labels and messages (3, 12, 20, 25).

Considering all the above, we hypothesized that sociodemographic environment and health status were related to population HL and that the latter could in turn be a determinant of health service use. Based on this assumption, the objectives of the present study were as follow: (a), to estimate the prevalence of inadequate or problematic health literacy and to analyze the relationship between this prevalence and the variables socioeconomic environment and health status; and (b), to analyze the association between HL and service use variables in the general population aged 15 and over in the Valencian Community.

## 2. Methods

Cross-sectional descriptive study based on a sample of 5,485 subjects, representative of the non-institutionalized population aged 15 or over, participating in the Health Interview Survey of the Valencian Community 2016 (HISVC-2016).

The HISVC-2016 data collection was carried out between May and December 2016, on a sample of 5,280 dwellings, identified from the Population Information System of the Regional Ministry of Universal Health and Public Health. The total sample size was 7,888 subjects in 2016, of which 5,485 were adults and 2,403 under 15 years of age residing in the Valencian Community, an autonomous community with almost five million inhabitants in 2016. The sample subjects were selected using a complex sampling design that assigned each subject a weighting according to their representativeness. The weights were included in the HISVC-2016 databases provided by the Health Plan Service of the Conselleria de Sanitat of the Generalitat Valenciana (the Health Ministry of the Valencian Government). The information was collected by means of a personal interview at the respondent’s home, assisted by tablets and other mobile devices. The questionnaire was completed by the interviewer, with the answers provided by the selected adult. Details on the survey methodology (sample design, sample size, sampling procedure, consent, ethics, etc.) have been previously published (29).

An informed consent was required from every participant in the HISVC-2016 by the Valencian Health Authorities. According to national regulations, data from National or Regional Health Surveys are public in Spain and the Valencian Health Authorities are responsible for and guarantee confidentiality and anonymity, making the approval of an ethics committee unnecessary (30). The researchers only had access to public data that had been rendered anonymous, and so this research poses no ethical issues.

To estimate HL, we used the HLS-EU-Q16 Questionnaire (31) that evaluates HL comprehensively and rapidly. It presents a suitable level of understanding and satisfactory psychometric properties (21). In this sense, reliability was high, with an intraclass correlation coefficient de 0.923 and a kappa of 0.814. The factorial analysis suggested a unifactorial structure with 79.1% of variability explained by the common factor, with high factorial loads. Consistency was also high, with Cronbach’s alpha values of 0.982. For the collection of the information on the Health Literacy module, answer support cards were used with information on the answers of the HLS-EU-Q16 questionnaire (1-very easy, 2-easy, 3-difficult, 4- very difficult), which were also included in the computer support itself.

The “literacy level” variable was constructed based on the 16 items in the questionnaire. We only used the data of the 5,148 subjects who validly answered at least 14 items, transforming each item into a dichotomous response: very difficult and difficult = 0; easy and very easy = 1. Each subject’s final score corresponded to the sum of the scores (0 or 1) of the 16 items. A score between 0 and 8 points was considered to represent an ‘inadequate level’, between 9 and 12 points, a ‘problematic level’, and between 13 and 16 points, a ‘sufficient level’. For the association analyses and the multivariate regression models, the variable was dichotomized into ‘Inadequate or problematic literacy’ (a score between 0 and 12) and ‘Sufficient literacy’ (a score between 13 and 16) (32). The standardized literacy index (SLI) calculated as (Mean-1)*(16/3) was calculated, with Mean = arithmetic mean of the scores (from 1 to 4) of the items answered. This index varied between 0 (lowest literacy) and 16 (highest literacy). The SLI behavior was characterized using established cut-off points and according to the sociodemographic characteristics of the health status of the population aged 15+ years.

The following sociodemographic variables were analyzed: sex (man, woman), age (15–24; 25–39; 40–64; 65–84; 85 and over), maximum level of studies attained (no studies, primary, secondary, university), employment status (working, unemployed, retired, studying, other situation), country of birth (Spain, other), income (<600, 600–1,200, 1,200–1,800, 1,800–2,700, >2,700 euros of monthly net income). The health status variables considered were: Self-perceived health (very good, good, fair, bad, very bad. For the analysis, it was classified as very good or good corresponded to ‘Good’; and fair, bad or very bad corresponded to ‘Bad’), chronic disease (yes/no) and activity limitations in the last 6 months, measured by the Global Activity Limitation Indicator – GALI (severely limited, limited but not severely, not at all limited). The following variables relating to service use were studied: medicine consumption (consumption over the last 2 weeks: yes/no); or use of health services (use of any service in the last 12 months: yes/no); consulted a general practitioner (any consultation over the last 4 weeks: yes/no); consulted a specialist (any consultation in the last 4 weeks: yes/no); need for care not met (no care received in the last 12 months: yes/no); knowledge and use of new health technologies (do you know of any health technology service: yes/no, and have you used one: yes/no); periodic preventive health check-ups (occupational, dentist, eye doctor: never or more than 3 years ago/less than 3 years ago); and preventive gynecological check-ups, mammography or cytology, only in women (never or more than 3 years ago/less than 3 years ago).

### 2.1. Statistical analysis

The percentages of inadequate or problematic literacy were calculated first, together with the SLI means, both globally and according to studied variable categories with 95% confidence intervals (CI95%). The Chi-square test was performed to analyze the relationship between inadequate or problematic literacy and demographic, socioeconomic and health status variables. Student’s t-test and ANOVA were performed to analyze differences in mean SLI values according to demographic, socioeconomic and health status categories. Bivariate and multivariate logistic regression models were adjusted to estimate the crude and adjusted odds ratios (ORs) of association between the dichotomous variable ‘Inadequate or problematic literacy /Sufficient literacy’ and the rest of the variables.

To analyze the association between HL and service use variables, we calculated the percentages of the variable categories according to each HL category (inadequate or problematic literacy/sufficient HL) and for the total, with 95% confidence intervals (CI95%).

Bivariate and multivariate logistic regression models were adjusted in order to estimate the crude and adjusted odds ratios (and 95% confidence intervals) of association between service use and the rest of variables.

In all estimates and models, the weights of the individuals in the sample were used according to the complex sample design. We employed IBM SPSS® software to perform all the calculations and a 0.05 significance level of reference.

## 3. Results

Table 1 shows the percentages of inadequate or problematic literacy as well as the average health literacy index values according to different categories of the sociodemographic and health status explanatory variables. We found that in the Valencian Community, 12.8% of the surveyed subjects in the general population presented inadequate or problematic HL levels (12.5% of women and 13.0% of men). Certain population groups presented higher degrees of inadequate or problematic HL: people aged 85 years or over (63.1%); with a low level of education (46.5%); retired people (27.4%); people in other employment situations (25.0%); foreigners (18.1%); and low-income people (16.2%). They presented significant differences in all variables. In the same way, HL was worse among those who perceived their health status as poor (26.9%), had a chronic disease (18.5%), or activity limitations (56.4% severe, 19.7% not severe), again presenting significant differences (*p* < 0.05) in all variables.

**Table 1:** Percentages of inadequate or problematic HL and mean values of the standardized literacy index (95% CI), according to categories of the explanatory sociodemographic and health status variables.

Variable	Subjects	Inadequate or problematic HL			Standardized literacy index		
	*n*	%	95% CI		Mean values	95% CI	
			*LL*	*UL*		*LL*	*UL*
Total sample	5,148	12.8	11.9	13.7	11.84	11.76	11.92
Sociodemographic variables							
Age**							
15-24	584	5.7	3.8	7.6	12.52	12.31	12.72
25–39	1,305	7.4	6.0	8.8	12.73	12.59	12.87
40–64	2,199	9.1	7.9	10.3	11.96	11.84	12.07
65–84	918	25.9	23.1	28.7	10.54	10.34	10.75
85 or more	141	63.1	55.1	71.1	7.37	6.63	8.10
Sex*							
Male	2,503	12.5	11.2	13.8	11.94	11.82	12.05
Female	2,646	13.0	11.7	14.3	11.75	11.63	11.86
Level of education**							
No studies	497	46.5	42.1	50.9	8.99	8.65	9.32
Primary	1,344	15.6	13.7	17.5	11.18	11.03	11.33
Secondary	2,133	6.6	5.5	7.7	12.25	12.15	12.36
University	1,168	6.4	5.0	7.8	13.06	12.90	13.21
Employment status**							
Working	2,152	7.6	6.5	8.7	12.45	12.34	12.55
Unemployed	900	6.2	4.6	7.8	11.81	11.64	11.98
Retired	1,030	27.4	24.7	30.1	10.52	10.31	10.72
Studying	529	5.5	3.6	7.4	13.25	13.03	13.46
Others	509	25.0	21.2	28.8	10.55	10.24	10.86
Country of birth**							
Spain	4,475	12.0	11.0	13.0	11.91	11.82	12.00
Other	673	18.1	15.2	21.0	11.35	11.15	11.56
Income**							
< 600	406	15.3	11.8	18.8	11.28	10.96	11.59
600–1,200	1,644	16.2	14.4	18.0	11.49	11.34	11.64
1,200–1800	1,043	9.7	7.9	11.5	11.90	11.73	12.07
1800–2,700	668	12.6	10.1	15.1	12.18	11.97	12.38
> 2,700	436	4.8	2.8	6.8	12.64	12.38	12.89
Health status variables							
Self-perceived health**							
Good	3,787	7.7	6.9	8.5	12.32	12.24	12.40
Bad	1,361	26.9	24.5	29.3	10.49	10.30	10.68
Chronic disease**							
No	2,686	7.4	6.4	8.4	12.44	12.34	12.53
Yes	2,460	18.5	17.0	20.0	11.19	11.06	11.31
Activity limitation**							
Severely limited	283	56.4	50.6	62.2	8.38	7.83	8.93
Limited but not severely	914	19.7	19.7	17.1	11.29	11.08	11.49
Not at all limited	3,950	8.0	7.2	8.8	12.21	12.13	12.29

The total number of subjects were those who answered 14 or more items of the literacy questionnaire. HL, Health Literacy; SLI, Standardized Literacy Index; CI, Confidence Interval; LL, lower limit; UL, upper limit. ^*^Statistically significant variables, *p* < 0.05, based on the difference of the SLI mean values according to categories, established by Student’s t-test and ANOVA. ^**^Statistically significant variables, *p* < 0.05, based on the differences in percentages and mean values of SLI according to categories, established by the chi square test, the Student’s *t*-test and ANOVA.

When adjusting a multivariate model, with HL (inadequate or problematic literacy vs. sufficient literacy) as a response variable and socioeconomic and health status variables as explanatory variables (Table 2), all presented a significant association except chronic disease.

**Table 2:** Adjusted odds ratio and 95% CI association between health literacy (inadequate or problematic vs. sufficient literacy category) and other demographic, socioeconomic, and health variables.

Variable	Adjusted OR	95% CI		*p*
		LL	UL	
Age				<0.001*
15-24	1.00			
25–39	1.36	0.60	3.06	0.460
40–64	1.01	0.45	2.29	0.977
65–84	2.64	1.07	6.52	0.035*
>85	8.28	3.11	22.07	<0.001*
Sex				0.002*
Female	1.00			
Male	1.43	1.15	1.80	0.002*
Educational level				<0.001*
University	1.00			
No studies	4.44	2.92	6.74	<0.001*
Primary	1.73	1.19	2.50	0.004*
Secondary	0.84	0.59	1.20	0.337
Employment status				<0.001*
Working	1.00			
Unemployed	0.70	0.47	1.04	0.074
Retired	0.91	0.56	1.46	0.694
Studying	0.33	0.13	0.81	0.015*
Others	1.75	1.19	2.58	0.004*
Country of birth				<0.001*
Spain	1.00			
Other country	5.01	3.76	6.68	<0.001*
Income				<0.001*
> 2,700 euros	1.00			
<600 euros	1.60	0.86	2.96	0.137
600 to 1,200 euros	1.86	1.09	3.16	0.023*
1,200 to 1,800 euros	1.69	0.96	2.94	0.065
1,800 to 2,700 euros	3.20	1.83	5.61	<0.001*
Self-perceived health				0.028*
Good	1.00			
Bad	1.38	1.04	1.84	0.028*
Activity limitation				<0.001*
Not at all limited	1.00			
Severely limited	8.98	6.19	13.03	<0.001*
Limited but not severely	2.11	1.60	2.79	<0.001*

CI, Confidence Interval; LL, lower limit; UL, upper limit; OR, Odds Ratio. ^*^Significant differences.

An older man (65–84 years), with no higher education, retired, born outside Spain, with an income between 600 and 1,200 euros a month, with poor self-perceived health and functional limitation, would be highly likely of presenting inadequate or problematic literacy – with an estimated probability per model of 0.84762. On the other hand, a working and university-educated young woman (aged 25–39 years) born in Spain, earning over 2,700 euros a month, with good self-perceived health and no functional limitation would have a low probability of presenting inadequate or problematic literacy – with an estimated probability per model of 0.01794.

Regarding the variables related to medicine consumption, service use and preventive practices, subjects with inadequate or problematic HL presented: lower medicine consumption (10.0% vs. 32.1%); a greater use of health services in the last 12 months (95.1% vs. 84.8%); a higher percentage of general practitioner visits (39.5% vs. 20.8%) and specialist visits (18.0% vs. 12.2%), lower levels of knowledge (44.5% vs. 73.9%) and use (24.7% vs. 44.7%) of new health technologies and less preventive occupational, dentist and eye doctor check-ups (21.5% vs. 7.9%) as well as less gynecological visits (48.1% vs. 18.6%) with significant percentage differences (*p* < 0.05) – see Table 3.

**Table 3:** Percentages and confidence intervals (95%) according to HL categories (inadequate or problematic HL, sufficient) of various results regarding medicine consumption, knowledge of new technologies, and use of health and preventive services.

Variable	Inadequate or problematic HL (*n* = 656)			Sufficient HL (*n* = 4,492)			Total (*n* = 5,148)		
	%	95% CI		%	95% CI		%	95% CI	
		*LL*	*UL*		*LL*	*UL*		*LL*	*UL*
Medication* (Consumed over the last 2 weeks)	10.0	7.7	12.3	32.1	30.7	33.5	29.3	28.1	30.5
Use of health services* (Use of one in the last 12 months)	95.1	93.4	96.8	84.8	83.8	85.8	86.1	85.2	87.0
Consulted a GP* (Any visit over last 4 weeks)	39.5	35.8	43.2	20.8	19.6	22.0	23.1	21.9	24.3
Consulted a specialist* (Any visit over last 4 weeks)	18.0	15.1	20.9	12.2	11.2	13.2	13.0	12.1	13.9
Care need not met (Care not received last 12 months)	4.3	2.7	5.9	3.0	2.5	3.5	3.2	2.7	3.7
New technology services* (knows some)	44.5	40.7	48.3	73.9	72.6	75.2	70.1	68.8	71.4
New technology services* (used some)	24.7	21.4	28.0	44.7	43.2	46.2	42.2	40.9	43.5
Periodic preventive check-ups: occupational, dentist, eye doctor* (Never or more than 3 years ago)	21.5	18.4	24.6	7.9	7.1	8.7	9.6	8.8	10.4
Gynecology preventive check-ups, mammography or cytology (Never or more than 3 years ago, only women)*	48.1	42.8	53.4	18.6	17.0	20.2	22.4	20.8	24.0

CI, Confidence Intervals; HL, Health Literacy; LL, lower limit; UL, upper limit. *Significant differences (*p* < 0.05) established by the chi square test.

Table 4 summarizes the association of the variables relating to services use (as outcome variables), including the use of preventive and general health services, medicine consumption, and knowledge and management of new technologies with HL variable (as explanatory variable). The results both of the simple analysis and adjusting for sociodemographic variables and health status revealed that people with inadequate or problematic HL were: 1.66 times more likely to use health services; 1.43 times more likely to consult a general practitioner; 1.41 and 2.08 times more likely of not having preventive health check-ups (including occupational, dentist, eye doctor visits as well as gynecological, mammography or cytology check-ups in the case of women). However, it was observed that inadequate HL could explain a lower probability of taking medication (OR 0.43) and poorer knowledge of new health technologies (OR 0.53).

**Table 4:** Simple and adjusted odds ratio of several variables of use of preventive and health services, knowledge and management of new technologies with HL (inadequate or problematic), adjusting for sociodemographic variables and health status.

Variable response	Simple OR	OR adjusted sociodemographic variables^1^	OR adjusted sociodemographic and health variables^2^
Medication use (last 2 weeks)	**0.23** (0.17–0.31)	**0.37** (0.27–0.52)	**0.43** (0.30–0.61)
Use of health services (last 12 months)	**2.72** (1.86–3.96)	**2.19** (1.45–3.29)	**1.66** (1.09–2.54)
Consulted a GP (any visit over last 4 weeks)	**2.47** (2.04–2.98)	**1.86** (1.50–2.32)	**1.43** (1.13–1.80)
Consulted a specialist (any visit over last 4 weeks)	**1.50** (1.17–1.91)	**1.50** (1.13–1.99)	0,94 (0,69-1,28)
Care need not met (last 12 months)	1,19 (0,73-1,93)	**2.07** (1.19–3.57)	1,31 (0,73-3,35)
New technology services (knows some)	**0.23** (0.19–0.28)	**0.55** (0.43–0.69)	**0.53** (0.42–0.67)
New technology services (has used some)	**0.37** (0.30–0.46)	0,87 (0,67-1,12)	0,82 (0,63-1,06)
Preventive occupational, dentist, eye doctor check-ups (never or more than 3 years ago)	**2.67** (2.10–3.38)	**1.44** (1.09–1.92)	**1.41** (1.05–1.88)
Gynecology preventive check-ups, mammography or cytology (women only, never or more than 3 years ago)	**4.51** (3.46–5.89)	**2.16** (1.52–3.07)	**2.08** (1.44–3.01)

CI, Confidence Interval; OR, Odds Ratio; HL, Health Literacy. In bold *p* < 0.05. ^1^Age, sex, education, employment status, country of birth, income. ^2^Self-perceived health, chronic disease, activity limitations.

The Supplementary material presents the complete final model of the association between HL and different variables of use of preventive and health services, knowledge and management of new technologies, adjusted to socio-demographic variables and health status for the total population.

## 4. Discussion

The present study centered on the adult population of the Valencian Community (Spain) and described HL according to categories of socioeconomic variables, health status, and use of health services. The results showed a significant association between HL and variables relating to service use and the use of new health technologies.

Although the global percentage of inadequate or problematic health literacy did not seem to be high (12.8%), some population subgroups presented values above 60% (for example, those aged above 85 years). Generally, the percentages of people with inadequate or problematic HL levels were lower than the percentages reported in other studies – i.e., between 28 and 66% of people with problematic HL levels (9, 28, 33–36).

In Europe, described levels of limited HL were higher – based on the HLS-EU Q47 (the long version of the questionnaire) –, the European average reaching 47.6% (8, 18). The country with the lowest reported levels of limited HL is the Netherlands, with 28.7% – still well above the levels found for the Valencian Community. The results in this work for Spain showed the highest percentages (together with Bulgaria) of populations with limited HL, accounting for around 58.3% (20) However, a study conducted in another Spanish region described values similar to ours, including a 15.4% rate of limited HL (10.3% inadequate literacy and 5.1% problematic literacy), which was also measured using the HLS-EU Q16 questionnaire (23).

HL level differences between countries have been explained both by contextual circumstances and individual traits (13, 20). In other words, HL depends on individual abilities and context-specific demands and expectations, determined by variables such as health culture, health care system complexity, the history of media education, information campaigns, as well as the contents of national and regional health policies. In this sense, in Spain’s case of a public and universal health system, this lower reported percentage of limited HL in the general population of the Valencian Community could perhaps be due to the population sample characteristics. Such features may include, for example: a population with high educational levels and young people; the type of information collected (the questionnaire was not self-administered so functional literacy was not assessed); a social desirability bias due to the interviewer’s presence; highly diligent interviewers who explained each questionnaire item; the participation of people with extensive health system knowledge and experience of health institutions; people who perceive low complexity in the access and use of health services in the region; a positive perception of personal competencies or skills; and populations that have participated in health education programs, among others. In this way, it would be important to assess the impact of intervention policies on self-care improvement promoted within the Valencian Community’s health strategy framework, such as attention to chronicity or active aging.

Regarding the HLS-EU-Q16 instrument, it should be noted that despite its extensive use (11, 28, 36–38), a recently published study questioned the representativeness of the underlying conceptual model used to measure HL (13). Another population study conducted in Spain (in the Mediterranean region of Catalonia, close to the Valencian Community) indicated that the substantial differences found may indicate a limitation specific to each country, which could be affecting the accuracy of the short HLS-EU-Q16 questionnaire (23). It would thus be necessary to further explore these result differences, as well as other variables associated with HL in the Valencian Community. The concepts underlying the measurements of an instrument based on a subjective, self-reported evaluation should also be examined (39). Moreover, an assessment of functional skills relating to health literacy could also be included, in addition to the self-declared/reported ability collected by the HLS-EU Q16 questionnaire.

Overall, the literature has shown that limited literacy follows a social gradient and may further accentuate existing inequalities (4–6, 10, 25, 36). Our results support the literature, which shows a significant relationship between HL and older age, low educational levels, low perceived social status, lower income, and migratory status, among others (3, 5, 6, 10). Moreover, most studies have found an association between limited HL and health status, mostly relating to self-perceived poor health, having a chronic disease and activity limitations (3, 10, 12, 20, 25). A study showed that in the Spanish population, age, level of education, and self-perceived health were the three main predictors of HL (measured with the HLS-Q47) (22). The study carried out in the Catalan population found that the factors educational level, socioeconomic status and physical limitations contributed the most to insufficient HL (measured using the HLS-EU-Q16) (23).

Regarding medicine consumption, subjects with insufficient HL presented lower levels of medication consumption. In this sense, evidence was found that a low HL level was related to worse skills in the appropriate taking of medication, worse performance in the use of dosing instruments, a lesser probability of identifying prescribed medications or a misinterpretation of labeling (3). It therefore showed that low literacy also leads to more negative experiences and errors in treatments and medication use. On the other hand, the evidence regarding adherence to medication treatments and procedures is not entirely clear (12, 40, 41).

With respect to service use, our results showed that subjects with inadequate or problematic HL made a greater use of health services, general practitioner consultations and specialist consultations. This trend has been reported in other studies (3, 12, 20, 25). For example, results of the latest study conducted in several European countries (HLS19) have shown that the higher the HL, the lower the use of emergency services, the fewer the contacts with GPs/family doctors, medical or surgical specialists, as well as short inpatient hospital services, and day patient hospital services (25).

However, when analyzing preventive practices, the results showed fewer preventive screenings among people with low HL. In this sense, the evidence shows less participation in health promotion programs and preventive programs, e.g., mammography screening and influenza immunizations (3, 5, 12, 42, 43). Concerning the use of Pap tests or colorectal cancer screening, the initial evidence was not entirely clear, although the latest studies point to the aforementioned association (3, 12, 43).

Our results showed poorer knowledge and use of new health technologies. Regarding the use of ICTs (information and communication technologies), some studies point to barriers relating to the use of websites, telephone interactions, and completion of health forms, etc. –which complicates the requirements to ensure successful decision-making by the low-HL (10, 44) In this sense, a series of recommendations have been proposed to promote better access and use of health information and therefore to improve HL (10, 44). In Spain, a study validated the eHealth questionnaire, which is a scale on the aptitude to use eHealth (45), that is, to measure digital literacy in health, which can be considered to specifically assess this dimension.

HL is an indicator of a population’s degree of competence to responsibly manage their own health. Greater HL levels could translate into a greater competence (44), and thus increase the probability of carrying out preventive practices, improving the use of the health system. The HL could, therefore, be a predictor variable of the use of services and the use of new health technologies.

In summary, the HL could act as a mediator of health outcomes and service use and new technologies. HL would thus behave similarly to the “self-perceived health” indicator. The latter is widely studied and recognized as a predictor of various health outcomes and service use (46, 47) However, unlike this indicator, the HL would behave as a potentially modifiable mediator, thanks to health education policies, health programs, or citizen empowerment, aimed at improving health equity and reducing or eliminating inequalities.

### 4.1. Strengths and limitations

The present work was a cross-sectional study, which makes it difficult to draw causal conclusions. On the other hand, a standardized instrument (HLS-EUQ16) that has shown to present adequate comprehension and good psychometric properties was used. This validated instrument has proven to be a simple and reliable way to collect population HL information. The present study was based on a large, representative sample of the population of a Spanish region. These data (collected for the first time in the Valencian Community’s population), can be used as a starting point for follow-up studies and analyses of the important role of HL. Indeed, inequalities have increased significantly following the measures adopted to face the crisis and pandemic. In this study, we only used the category classification suggested in other studies (inadequate/problematic and sufficient). The full potential of continuous scoring and other literacy scale categories is worth exploring in the future.

## 5. Conclusion

Health literacy levels of the population of the Valencian Community (Spain) were measured. Percentages of inadequate or problematic HL differed according to the population group. The Valencian Community’s population presents an inadequate or problematic level of health literacy which is slightly below the European and Spanish average. A specific population profile with an inadequate or problematic HL level was identified. This profile should be regarded as a target group for HL improvement measures, including the implementation of specific policies and programs. The ultimate objective would thus be to reduce health disparities and to help to improve their health status.

HL was significantly associated with variables relating to service use and the use of new health technologies, both in the simple analysis and when adjusting for various demographic, socioeconomic environment and health status variables. Improving HL could improve the levels of use both of health services and of new health technologies.

## Data availability statement

The data analyzed in this study is subject to the following licenses/restrictions: The data of the Health Interview Survey of the Valencian Community 2016 analyzed for this study can be obtained from the Office of Health Plan (Conselleria de Sanitat of the Valencian Autonomous Government) on demand and by personal registration: https://www.sp.san.gva.es/suscripciones/inicioSuscripciones.jsp?menuRaizPortal=SANMS&Opcion=SANPS9I&MenuSup=SANMS&perfil=inst. Requests to access these datasets should be directed to https://www.sp.san.gva.es/suscripciones/inicioSuscripciones.jsp?menuRaizPortal=SANMS&Opcion=SANPS9I&MenuSup=SANMS&perfil=inst.

## Ethics statement

Ethical review and approval was not required for the study on human participants in accordance with the local legislation and institutional requirements. Written informed consent for participation was not required for this study in accordance with the national legislation and the institutional requirements.

## Author contributions

AN and NT-F designed the protocol, led the project, and wrote the first draft. CB, RM, and M^a^I collected the necessary data. AN and PP-Z performed the statistical analysis. All authors providing critical comments, read, and approved the final manuscript.

## Abbreviations

HL: Health literacy
SLI: Standardized literacy index
HISVC: Health interview survey of the Valencian community
HLS-EU-Q16: European health literacy survey questionnaire
GALI: Global activity limitation indicator
