# Supplementary material for: Health literacy: association with socioeconomic determinants and the use of health services in Spain
Source: Front Public Health. 2023 Oct 12;11:1226420. doi: 10.3389/fpubh.2023.1226420 (PMC10602755; doi:10.3389/fpubh.2023.1226420)
Supplement: Supplementary file 1 [file Table_1.pdf]

Supplementary Material

Health literacy: Association with socioeconomic determinants and the use of health services in Spain

Nayara Tamayo-Fonseca[1], Pamela Pereyra-Zamora[1*], Carmen Barona[2,3,4], Rosa Mas[2,3], Mª Ángeles Irles[2], Andreu Nolasco[1]

*** Correspondence:** Corresponding Author: pamela.pereyra@ua.es

Supplementary Table S1.

Adjusted odds ratio of association between HL (inadequate or problematic) and the variables: use of preventive and health services, knowledge and management of new technologies, sociodemographic and health status.

Variables	Medicine consumption			Use of services			General practitioner consultation			Specialist consultation			Care need not met			Preventive occupational check-ups			Gynaecological preventive check-ups (women)			Knows new technologies services			Uses a new technology service		
	OR	P	95% CI	OR	P	95% CI	OR	P	95% CI	OR	P	95% CI	OR	P	95% CI	OR	P	95% CI	OR	P	95% CI	OR	P	95% CI	OR	P	95% CI
LITERACY	*			*			*			**			**			*			*			*			**		
Sufficient	1.00			1.00			1.00			1.00			1.00			1.00			1.00			1.00			1.00		
Inadequate/problem	0.43	<0.001	0.30-0.61	1.66	0.018	1.09-2.54	1.43	0.003	1.13-1.80	0.94	0.687	0.69-1.28	1.31	0.364	0.73-2.35	1.41	0.021	1.05-1.88	2.08	<0.001	1.44-3.01	0.53	<0.001	0.42-0.67	0.82	0.135	0.63-1.06
SEX	*			*			*			**			*			*						**			*		
Women	1.00			1.00			1.00			1.00			1.00			1.00						1.00			1.00		
Men	1.29	0.002	1.10-1.51	0.43	<0.001	0.35-0.53	0.82	0.013	0.70-0.96	0.82	0.052	0.67-1.00	0.60	0.012	0.41-0.90	1.52	<0.001	1.22-1.90				0.87	0.088	0.74-1.02	0.80	0.003	0.70-0.93
AGE	*			*			*			**			*			*			*			*			*		
≥85	1.00			1.00			1.00			1.00			1.00			1.00			1.00			1.00			1.00		
15-24	16.18	<0.001	3.43-76.42	3.33	0.025	1.17-9.51	1.81	0.071	0.95-3.45	2.59	0.032	1.09-6.20	2.12	0.548	0.18-24.78	1.27	0.543	0.58-2.78	0.29	0.010	0.12-0.74	14.97	<0.001	7.04-31.85	6.12	<0.001	2.39-15.69
25-39	11.19	0.002	2.44-51.25	4.46	0.003	1.67-11.91	1.98	0.012	1.16-3.36	2.97	0.003	1.44-6.12	9.17	0.062	0.90-93.81	0.44	0.019	0.23-0.88	0.06	<0.001	0.03-0.13	9.51	<0.001	4.97-18.23	9.34	<0.001	3.82-22.86
40-64	8.11	0.007	1.79-36.78	3.41	0.012	1.31-8.89	1.33	0.251	0.82-2.18	2.39	0.011	1.23-4.67	5.99	0.123	0.62-58.38	0.76	0.365	0.42-1.38	0.03	<0.001	0.02-0.07	7.03	<0.001	3.80-13.02	5.28	<0.001	2.19-12.70
65-84	3.91	0.071	0.89-17.18	1.80	0.169	0.78-4.16	1.14	0.526	0.76-1.73	1.64	0.087	0.93-2.88	5.22	0.130	0.61-44.34	1.02	0.939	0.63-1.65	0.30	<0.001	0.16-0.55	3.85	<0.001	2.18-6.79	2.64	0.024	1.14-6.14
STUDIES	*			*			*			*			*			*			*			*			*		
University	1.00			1.00			1.00			1.00			1.00			1.00			1.00			1.00			1.00		
No studies	1.71	0.014	1.11-2.61	0.64	0.069	0.39-1.04	1.47	0.024	1.05-2.05	0.44	<0.001	0.29-0.66	0.39	0.057	0.15-1.03	2.78	<0.001	1.75-4.43	2.19	0.003	1.31-3.67	0.18	<0.001	0.13-0.26	0.29	<0.001	0.20-0.43
Primary	1.57	<0.001	1.23-2.01	0.59	0.001	0.43-0.80	1.47	0.003	1.14-1.90	0.29	<0.001	0.21-0.40	0.52	0.042	0.28-0.98	1.75	0.007	1.17-2.64	1.01	0.949	0.66-1.55	0.44	<0.001	0.34-0.58	0.52	<0.001	0.42-0.65
Secondary	0.98	0.878	0.80-1.21	0.97	0.802	0.73-1.27	1.46	0.001	1.16-1.84	0.49	<0.001	0.38-0.64	1.19	0.484	0.74-1.91	1.35	0.137	0.91-2.00	1.38	0.088	0.95-2.00	0.74	0.016	0.57-0.95	0.83	0.057	0.69-1.01
EMPLOYMENT SITUATION	*			*			*			*			*			*			*			*			*		
Working	1.00			1.00			1.00			1.00			1.00			1.00			1.00			1.00			1.00		
Unemployed	1.34	0.007	1.08-1.64	1.00	0.983	0.77-1.29	1.13	0.299	0.90-1.44	1.41	0.029	1.04-1.91	2.04	0.006	1.23-3.39	5.08	<0.001	3.58-7.22	1.82	0.004	1.22-2.74	0.87	0.228	0.69-1.09	0.70	<0.001	0.58-0.85
Retired	0.66	0.074	0.41-1.04	3.80	<0.001	2.06-6.99	2.12	<0.001	1.51-2.99	1.75	0.015	1.12-2.75	0.97	0.958	0.33-2.88	3.01	<0.001	1.84-4.91	0.97	0.920	0.52-1.82	0.66	0.017	0.46-0.93	0.47	<0.001	0.32-0.67
Studying	0.83	0.316	0.58-1.19	0.63	0.044	0.40-0.99	0.68	0.081	0.45-1.05	1.34	0.263	0.80-2.24	3.95	<0.001	1.86-8.42	1.11	0.772	0.56-2.18	3.04	<0.001	1.70-5.43	0.27	<0.001	0.18-0.42	0.48	<0.001	0.34-0.68
Others	0.70	0.053	0.49-1.01	2.48	0.001	1.42-4.33	1.88	<0.001	1.43-2.46	1.03	0.897	0.71-1.48	1.00	0.993	0.49-2.05	4.83	<0.001	3.18-7.33	1.55	0.089	0.94-2.58	0.65	0.003	0.49-0.87	0.38	<0.001	0.29-0.50
COUNTRY OF BIRTH	*			**			**			*			**			*			*			*			*		
Other country	1.00			1.00			1.00			1.00			1.00			1.00			1.00			1.00			1.00		
Spain	1.39	0.006	1.10-1.76	1.01	0.922	0.76-1.35	1.03	0.786	0.81-1.31	1.47	0.021	1.06-2.03	0.89	0.653	0.53-1.48	0.48	<0.001	0.35-0.65	0.61	0.006	0.42-0.87	2.31	<0.001	1.86-2.88	1.63	<0.001	1.32-2.00
INCOME	**			*			*			*			*			*			*			*			*		
> 2700 €	1.00			1.00			1.00			1.00			1.00			1.00			1.00			1.00			1.00		
< 600	0.88	0.524	0.61-1.29	0.46	0.001	0.29-0.72	0.84	0.339	0.59-1.20	0.52	0.003	0.34-0.81	3.15	0.053	0.99-10.09	9.14	<0.001	3.77-22.19	1.14	0.652	0.64-2.03	0.30	<0.001	0.20-0.46	0.40	<0.001	0.28-0.56
600 - 1200	0.92	0.532	0.70-1.21	0.74	0.107	0.52-1.07	0.64	0.003	0.48-0.86	0.48	<0.001	0.34-0.67	1.62	0.354	0.58-4.50	4.48	0.001	1.89-10.62	1.04	0.883	0.63-1.71	0.27	<0.001	0.18-0.38	0.37	<0.001	0.29-0.49
1200 - 1800	0.88	0.374	0.67-1.16	0.74	0.109	0.52-1.07	0.71	0.026	0.53-0.96	0.57	0.002	0.41-0.81	5.44	0.001	2.01-14.70	6.02	<0.001	2.52-14.38	1.39	0.213	0.83-2.32	0.58	0.005	0.40-0.85	0.73	0.020	0.56-0.95
1800 - 2700	1.20	0.212	0.90-1.59	1.19	0.387	0.80-1.77	1.11	0.495	0.82-1.51	0.44	<0.001	0.30-0.64	2.84	0.049	1.01-7.99	4.01	0.003	1.62-9.97	0.55	0.047	0.30-0.99	0.69	0.074	0.46-1.04	0.75	0.045	0.57-0.99
SELF-PERCEIVED HEALTH	*			**			*			*			**			*			**			**			*		
Bad	1.00			1.00			1.00			1.00			1.00			1.00			1.00			1.00			1.00		
Good	0.44	<0.001	0.32-0.59	1.15	0.452	0.80-1.66	1.24	0.040	1.01-1.53	2.77	<0.001	2.10-3.64	1.37	0.266	0.79-2.39	1.60	0.001	1.22-2.10	0.88	0.466	0.62-1.25	0.95	0.608	0.76-1.18	0.55	<0.001	0.44-0.68
CHRONIC DISEASE	*			*			*			*			*			*			**			**			**		
No	1.00			1.00			1.00			1.00			1.00			1.00			1.00			1.00			1.00		
Yes	0.25	<0.001	0.20-0.30	3.72	<0.001	2.85-4.87	2.01	<0.001	1.68-2.41	1.90	<0.001	1.47-2.44	0.43	0.001	0.26-0.70	0.66	0.002	0.50-0.86	0.86	0.315	0.65-1.15	1.02	0.860	0.84-1.23	0.96	0.608	0.81-1.13
GALI	*			*			*			*			*						*			*			*		
Not at all limited	1.00			1.00			1.00			1.00			1.00						1.00			1.00			1.00		
Severely limited	0.77	0.380	0.44-1.37	7.28	0.001	2.21-24.00	2.24	<0.001	1.63-3.10	2.49	<0.001	1.69-3.65	14.79	<0.001	7.62-28.71				1.85	0.032	1.05-3.25	1.34	0.124	0.92-1.95	2.27	<0.001	1.55-3.33
Limit not severely	0.53	<0.001	0.39-0.71	3.60	<0.001	2.28-5.67	1.81	<0.001	1.48-2.22	2.85	<0.001	2.22-3.66	6.26	<0.001	3.79-10.33				0.73	0.092	0.51-1.05	0.86	0.160	0.69-1.06	1.53	<0.001	1.23-1.91

Note. OR=Odds Ratio, HL= Health Literacy, CI= Confidence Interval, * Statistically significant ($p < 0.05$), ** Not significant
